# Comparative genomic analysis of esophageal squamous cell carcinoma among different geographic regions

**DOI:** 10.3389/fonc.2022.999424

**Published:** 2023-01-18

**Authors:** Ruixiang Zhang, Canjun Li, Zhiyi Wan, Jianjun Qin, Yong Li, Zhen Wang, Qingfeng Zheng, Xiaozheng Kang, Xiankai Chen, Yun Li, Jie He, Yin Li

**Affiliations:** ^1^ Department of Thoracic Surgery, National Cancer Center/National Clinical Research Center for Cancer/Cancer Hospital, Chinese Academy of Medical Sciences and Peking Union Medical College, Beijing, China; ^2^ Department of Radiation Oncology, National Cancer Center/National Clinical Research Center for Cancer/Cancer Hospital, Chinese Academy of Medical Sciences and Peking Union Medical College, Beijing, China; ^3^ Department of Medicine, Genecast Biotechnology Co., Ltd, Wuxi, China

**Keywords:** genomic characteristics, geographical differences, northern China, southern China, esophageal squamous cell carcinoma

## Abstract

**Introduction:**

Esophageal squamous cell carcinoma (ESCC) shows remarkable variation in incidence, survival, and risk factors. Although the genomic characteristics of ESCC have been extensively characterized, the genomic differences between different geographic regions remain unclear.

**Methods:**

In this study, we sequenced 111 patients with ESCC from northern (NC) and southern (SC) China, combined their data with those of 1081 cases from previous reports, and performed a comparative analysis among different regions. In total, 644 ESCC cases were collected from six geographic regions (NC, SC, Xinjiang, China [XJC], Japan [JP], Vietnam [VN], and Europe & America [EA]) as the discovery cohort. Validation cohort 1 included 437 patients with ESCC from the NC region. Validation cohort 2 included 54 and 57 patients from the NC and SC regions, respectively.

**Results:**

Patients with ESCC in different regions had different genomic characteristics, including DNA signatures, tumor mutation burdens, significantly mutated genes (SMGs), altered signaling pathways, and genes associated with clinical features. Based on both the DNA mutation signature and the mutation profile of the most common genes, the NC and SC groups were clustered close together, followed by the JP, XJC, EA, and VN groups. Compared to patients with ESCC from SC, SMGs, including *KMT2D*, *FAT1*, and *NOTCH1* were more frequently identified in patients with ESCC from NC. Furthermore, some genes (*TDG* and *DNAH8*) correlated with overall survival in completely opposite ways in patients with ESCC from different geographical regions.

**Conclusions:**

Our study provides insights into genomic differences in ESCC among different regions. These differences may be related to differences in environmental carcinogens, incidence, and survival.

## 1 Introduction

Esophageal squamous cell carcinoma (ESCC) is the predominant histological subtype of esophageal cancer and is characterized by marked geographic variations (1, 2). Approximately 70% of ESCC cases occur in China (3). In China, ESCC is the third most common cancer, with an estimated 477.9 thousand new cases occurring in 2015 (4). Furthermore, ESCC is particularly prevalent in specific geographic regions of China, such as Xinjiang Province, Chaoshan in southern China (SC), and the southern Taihang Mountains region (Yangcheng, Linxian, Cixian, and Shexian) in northern China (NC) (5–7).

Substantial differences are also present in the risk factors of ESCC among different geographical regions. Alcohol abuse and smoking increase the risk of ESCC in Western countries but represent minor factors in China (8, 9). The association between drinking and smoking and ESCC is stronger in Japan than in China (10). Food mutagens and nutritional deficiency may be major risk factors for ESCC in the Taihang Mountains region (5, 7). However, drinking hot tea is associated with an increased risk for ESCC in the Chaoshan region (6, 11, 12). The consumption of hot maté is a risk factor for South Americans (13, 14). For survival differences in ESCC, the overall mortality rate is also higher in China than in Japan (10). However, Chinese patients with ESCC have similar overall survival as American patients (15).

The development of ESCC depends on environmental factors, life-style, and genetic variation (10). Both endogenous processes and exogenous mutagenic exposures produce unique mutational signatures (16, 17). Therefore, understanding the genomic characteristics underlying the geographical variations in ESCC is necessary. Several studies have extensively characterized the genomic characteristics of ESCC (18–25); however, the genomic differences among different geographic regions remain unclear. Although recent studies have compared the genomic characteristics of ESCC in different countries (22, 26), the grouping of countries was too broad to distinguish between specific geographical units. In the present study, we performed a comparative genomic analysis of ESCC among different geographic regions by integrating 1081 ESCC cases from previous reports. A further 111 patients with ESCC from NC and SC were detected by target sequencing and used for validation.

## 2 Methods

### 2.1 Patients and data

Somatic mutation lists and clinical information of 1081 ESCC cases were obtained from published studies (18–25) or downloaded from the cBioPortal site (https://www.cbioportal.org/). In addition, data on 111 ESCC cases were extracted from our hospital and used as the validation set. The clinical characteristics of our cohort are presented in Additional File 1. All specimens were collected from patients who have given written informed consent for their samples to be used in scientific studies. This study was conducted in accordance with the Declaration of Helsinki and approved by the ethics committee of the Cancer Hospital Chinese Academy of Medical Sciences (20/388-2584).

### 2.2 Targeted sequencing

Tumor DNA and matched white blood cell DNA were extracted. The quantity and quality of isolated DNA were tested using a Qubit 3.0 fluorimeter (Life Technologies, Eugene, OR, USA). DNA libraries were constructed and captured using a targeted panel of 764 genes (Genecast Biotechnology Co., Ltd), according to the manufacturer’s protocol. Sequencing was performed using an Illumina NovaSeq 6000 platform (Illumina, Hayward, CA, USA). Clean reads were aligned to the reference genome (hg19) using Burrows-Wheeler Aligner (27). VarDict and FreeBayes were used to call single nucleotide variations and small insertions or deletions mutations, and these mutations were annotated using ANNOVAR. The final somatic mutations used for the following analyse were selected based on the following standards: (i) variant allele frequency ≥ 5%, (ii) not located in intergenic regions or intronic regions and no synonymous single nucleotide variations, and (iii) support reads ≥ 5. The genetic variants in our cohort are presented in Additional File 2.

### 2.3 Comparative mutational analyses

Patients were grouped based on geographic regions. Gene names reported in the different mutation lists were first harmonized using the HGNChelper package (28) in R version 4.1.0. The mutation data were then analyzed using the maftools package (29). Mutational signature analysis was performed using maftools and NMF packages (29, 30). The frequencies of the 96 single-nucleotide substitution patterns in each data set were visualized as three-dimensional bar plots. Genes with differences in mutation frequency were identified using the mafComapre function and visualized using the forestplot function in the maftools package. Clustering analyses were performed based on the Euclidean distance and average linkage method using the pheatmap package.

### 2.4 Statistical analysis

Statistical analysis was performed using the SPSS 22.0 (SPSS, Inc., Chicago, IL, USA) or R version 4.1.0. Fisher’s exact test was used to compare the differences in proportions between the two groups. The Wilcoxon test was used to compare tumor mutation burden (TMB). Survival analyses were performed using Kaplan-Meier curves and Cox regression analysis. Statistical tests were two-sided, and significance was set at a level of *P* < 0.05.

## 3 Results

### 3.1 Geographical differences in the somatic mutation landscape

**Figure 1:**
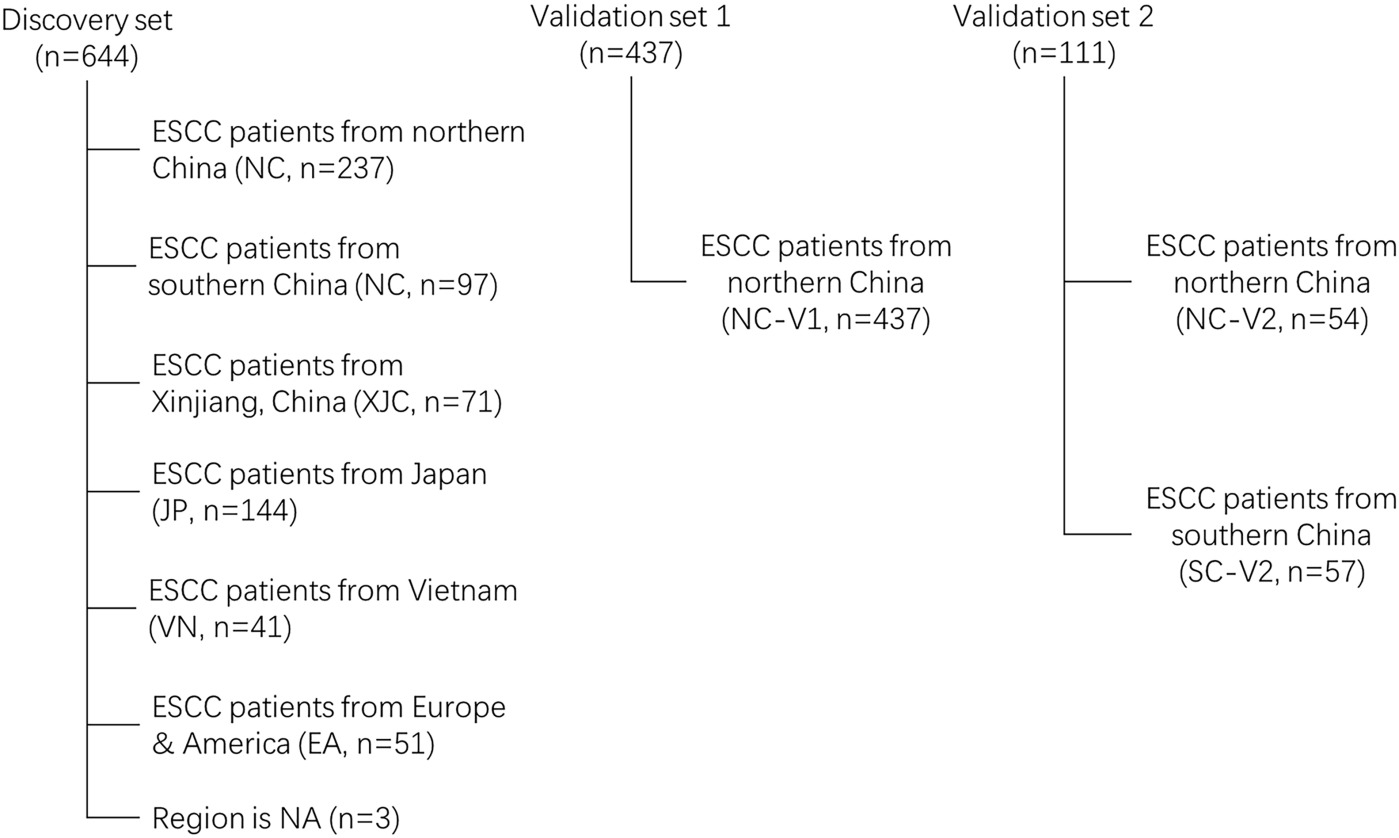
Flow chart of ESCC samples eligibility NC, northern China; SC, southern China; XJC, Xinjiang, China; JP, Japan; EA, Europe and America; VN, Vietnam. NA, not available; ESCC, esophageal squamous cell carcinoma.

**Figure 2:**
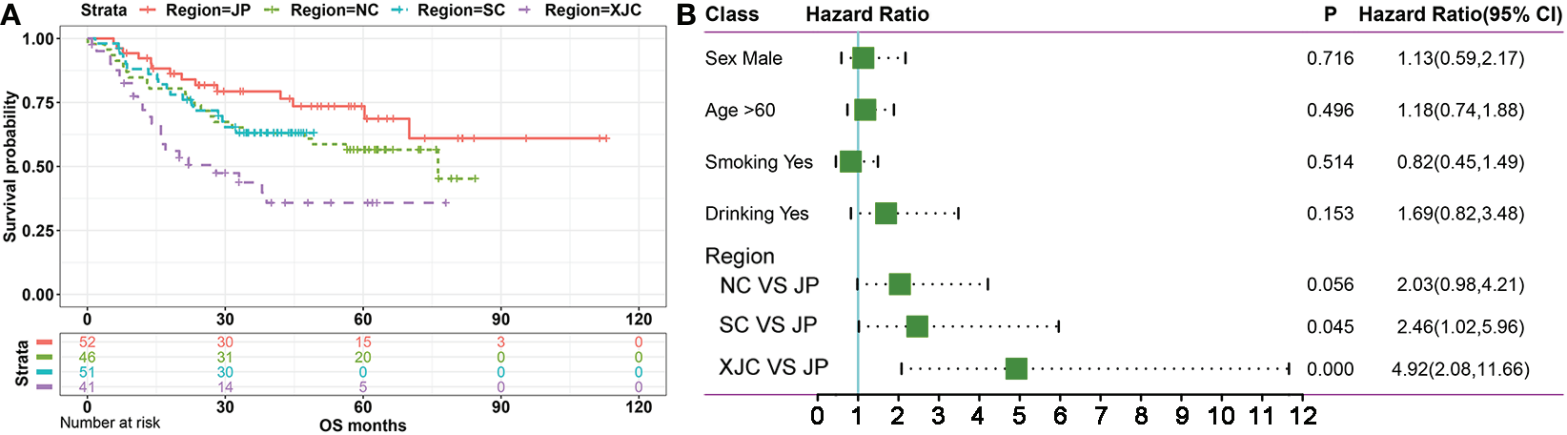
Survival analysis of patients with stage II ESCC from different regions. **(A)** Kaplan–Meier plots of overall survival in patients with stage II ESCC from different regions. **(B)** Mutivariate Cox regression analysis of overall survival in patients with stage II ESCC from different regions. NC, northern China; SC, southern China; XJC, Xinjiang, China; JP, Japan; OS, overall survival; ESCC, esophageal squamous cell carcinoma.

First, 644 ESCC cases were collected from eight previous reports (18–25) as a discovery cohort (**Figure 1**). These cases were divided into six groups based on geographic region: NC (most cases from the southern Taihang Mountains region), SC (most cases from the Chaoshan region), Xinjiang, China (XJC), Japan (JP), Vietnam (VN), and Europe and America (EA). Clinical information is presented in **Table 1**. The number of patients with stage II ESCC was high in all groups. Clinical information for four groups (NC, SC, XJC, and JP) was relatively complete. We then compared the survival of patients with stage II ESCC among the four groups. Patients in the NC and SC groups had similar overall survival (OS); however, those in the JP group had significantly better OS than those in the XJC group (**Figure 2A**, P = 0.004). Multivariate Cox analysis further showed that region was an independent prognostic factor for OS (**Figure 2B**). The significant differences in the survival of patients with ESCC by geographic region may be related to the differences in molecular characteristics among regions.

**Table 1:** Clinical information of patients with ESCC from different regions.

Region	NC	SC	XJC	JP	EA	VN
Sex						
Female	23	22	27	18	12	2
Male	214	66	44	126	39	39
Age						
>60	103	33	39	101	21	13
≤60	131	64	32	43	30	28
Stage						
I	62	4	7	9	6	0
II	53	60	47	52	22	31
III	120	33	17	71	18	9
IV	1	0	0	12	3	1
Smoking						
No	44	31	49	50	NA	NA
Yes	173	57	22	94	NA	NA
Drinking						
No	40	71	62	15	NA	NA
Yes	73	17	9	129	NA	NA

NC, northern China (most cases from the southern Taihang Mountains region); SC, southern China (most cases from the Chaoshan region); XJC, Xinjiang, China; JP, Japan; EA, Europe and America (including Russia, Ukraine, Canada, USA, and Brazil); VN, Vietnam; NA, not available; ESCC, esophageal squamous cell carcinoma.

**Figure 3:**
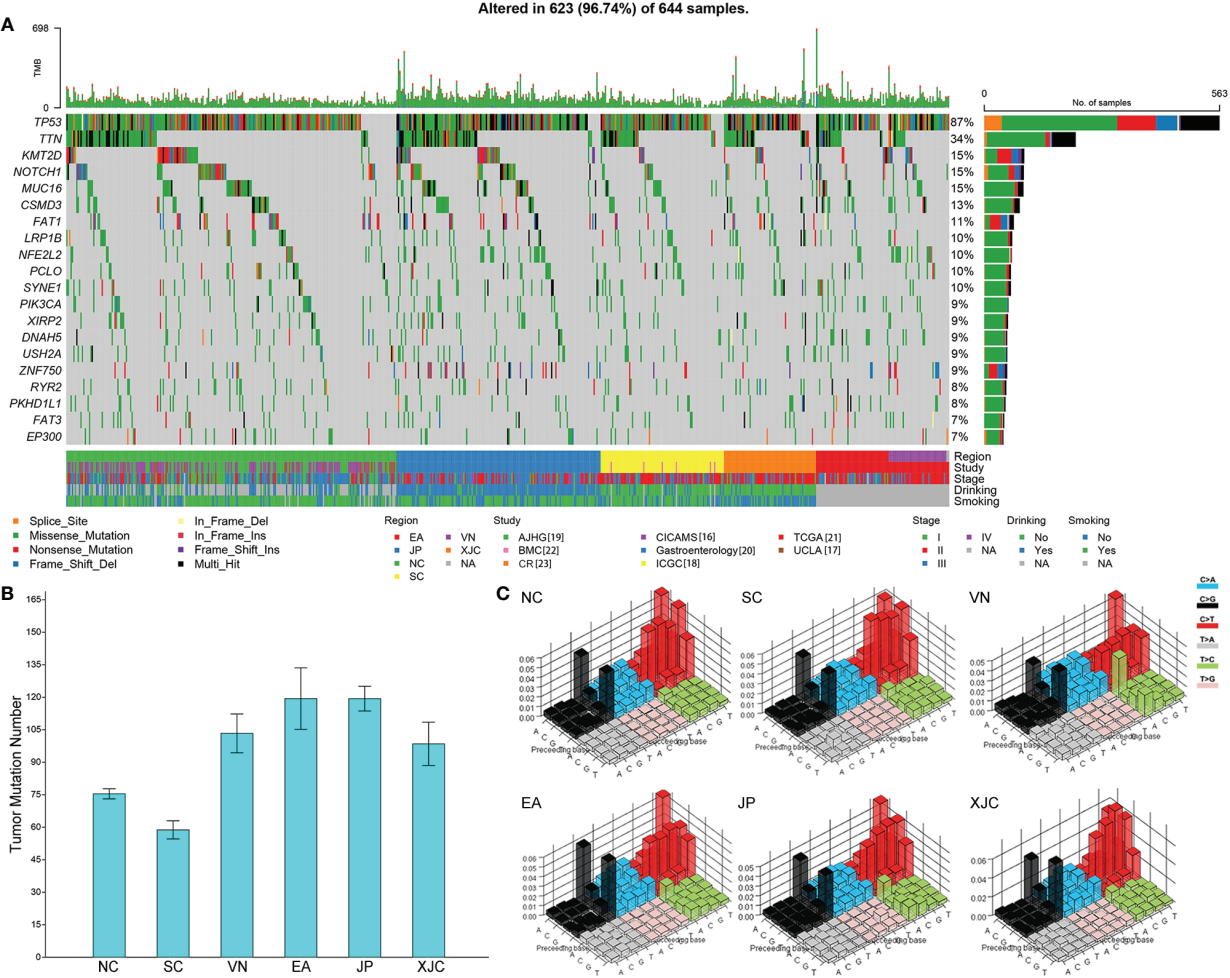
Geographical differences in the somatic mutation landscape. **(A)** Mutational landscape of all patients (top 20 genes). The genes were sorted by the mutation frequency, while samples were sorted by region. Comparison of tumor mutational burden **(B)** and mutation signature spectra **(C)** among different regions. Base substitutions are divided into 96 patterns based on mutation type and nucleotides flanking the mutated base. The height of the bar represents the proportion of each substitution pattern. NC, northern China; SC, southern China; XJC, Xinjiang, China; JP, Japan; EA, Europe and America; VN, Vietnam. NA, not available.

The somatic mutation landscape was then analyzed, and significant differences were observed in TMB of patients with ESCC among different geographical regions (**Figures 3A, B**). Tumor mutation burden was the lowest in the SC group, followed by the NC, XJC, and VN groups and was the highest in the EA and JP groups (**Figure 3B**). Although the overall mutation spectra were similar (signature COSMIC_1 and COSMIC_13) among different regions, ESCC cases in the XJC and VN groups showed relatively unique mutational characteristics (**Figure 3C**; **Table 2**). The unique mutation signature in the XJC group were COSMIC_3 (etiology: defects in DNA-DSB repair by HR) and COSMIC_6 (etiology: defective DNA mismatch repair), whereas the unique mutation signatures in the VN group were COSMIC_2 (etiology: APOBEC Cytidine Deaminase (C>T)) and COSMIC_4 (etiology: exposure to tobacco (smoking) mutagens). The proportion of APOBEC signature enriched ESCC was similar between the patients in each region, ranging from 58% to 68% (**Supplementary Figure 1A**). However, there were essentially no APOBEC signature-related genes shared among the groups (**Supplementary Figure 1B**). Only *ZNF750* was shared between the NC and JP groups and *RB1* was shared between the SC and JP groups (**Supplementary Figure 1B**).

**Table 2:** Most similar signatures of patients with ESCC from different regions.

Region	Most similar signature			
NC	COSMIC_1	COSMIC_13	COSMIC_5	
SC	COSMIC_13	COSMIC_5	COSMIC_1	
XJC	COSMIC_6	COSMIC_13	COSMIC_5	COSMIC_3
JP	COSMIC_13	COSMIC_1		
VN	COSMIC_4	COSMIC_2	COSMIC_5	
EA	COSMIC_1	COSMIC_13	COSMIC_5	

COSMIC_1, spontaneous deamination of 5-methylcytosine; COSMIC_2, APOBEC Cytidine Deaminase (C>T); COSMIC_3, defects in DNA-DSB repair by HR; COSMIC_4, exposure to tobacco (smoking) mutagens; COSMIC_5, Unknown; COSMIC_6, defective DNA mismatch repair; COSMIC_13, APOBEC Cytidine Deaminase (C>G). NC, northern China; SC, southern China; XJC, Xinjiang, China; JP, Japan; EA, Europe and America; VN, Vietnam; ESCC, esophageal squamous cell carcinoma.

**Figure 4:**
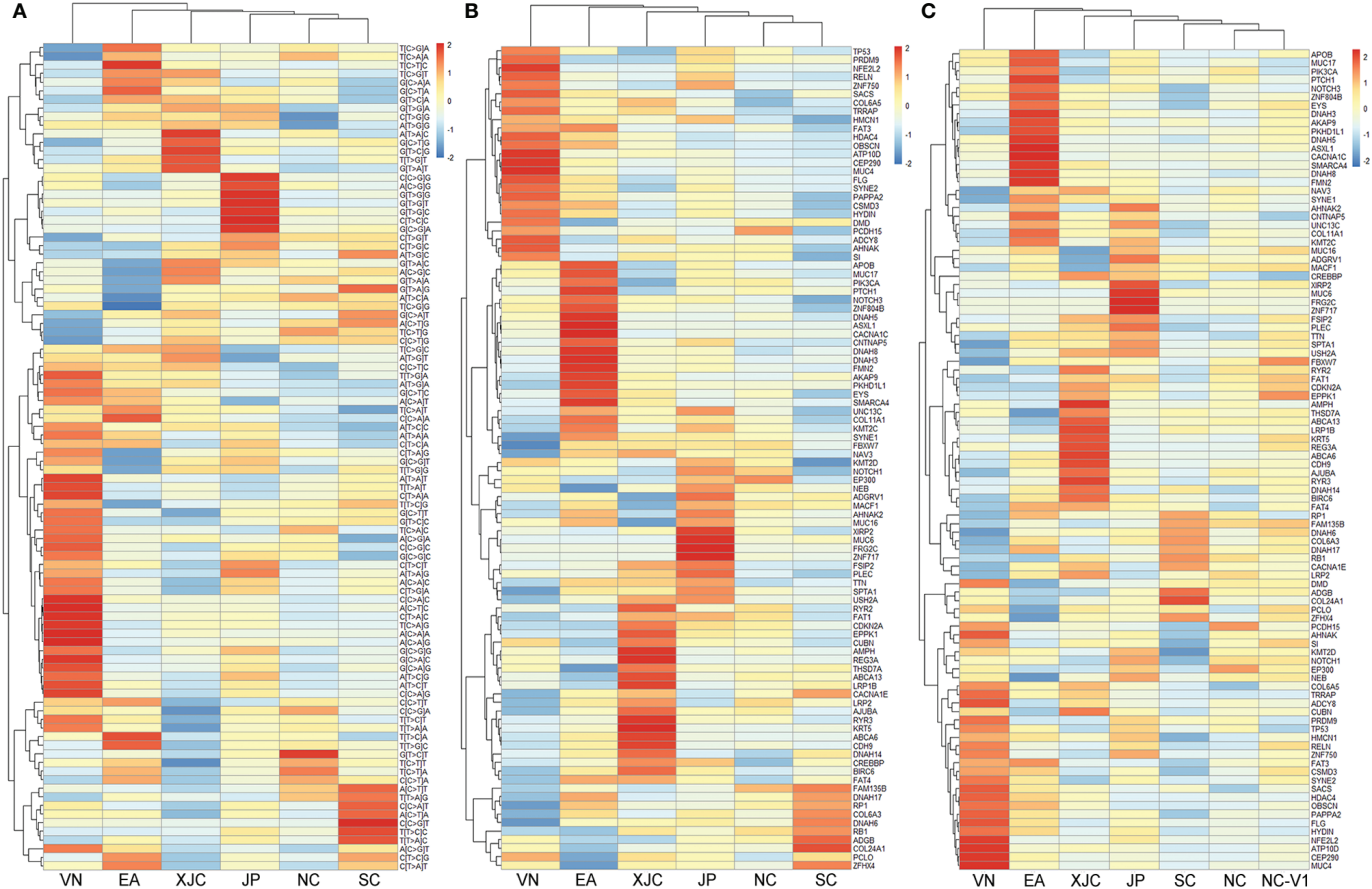
Clustering analysis of somatic mutations. **(A)** Clustering analysis of mutation signatures. Clustering analysis of top 30 mutant genes in the discovery set **(B)** and the validation set 1 **(C)**. NC, northern China; SC, southern China; XJC, Xinjiang, China; JP, Japan; EA, Europe and America; VN, Vietnam. NC-V1, northern China: validation set 1.

To investigate the relationship between the molecular characteristics of the regions, cluster analysis based on mutation spectra was performed. The NC and SC groups clustered close together, followed by the JP, XJC, EA, and VN groups (**Figure 4A**). Similar results were observed in the cluster analysis based on the union set of the top 30 mutant genes in each group (**Figure 4B**). A cohort of 437 ESCC cases (25) from NC (termed the NC-V1 group) was collected and used for validation. Cluster analysis showed that the NC-V1 group clustered with the NC group (**Figure 4C**).

**Figure 5:**
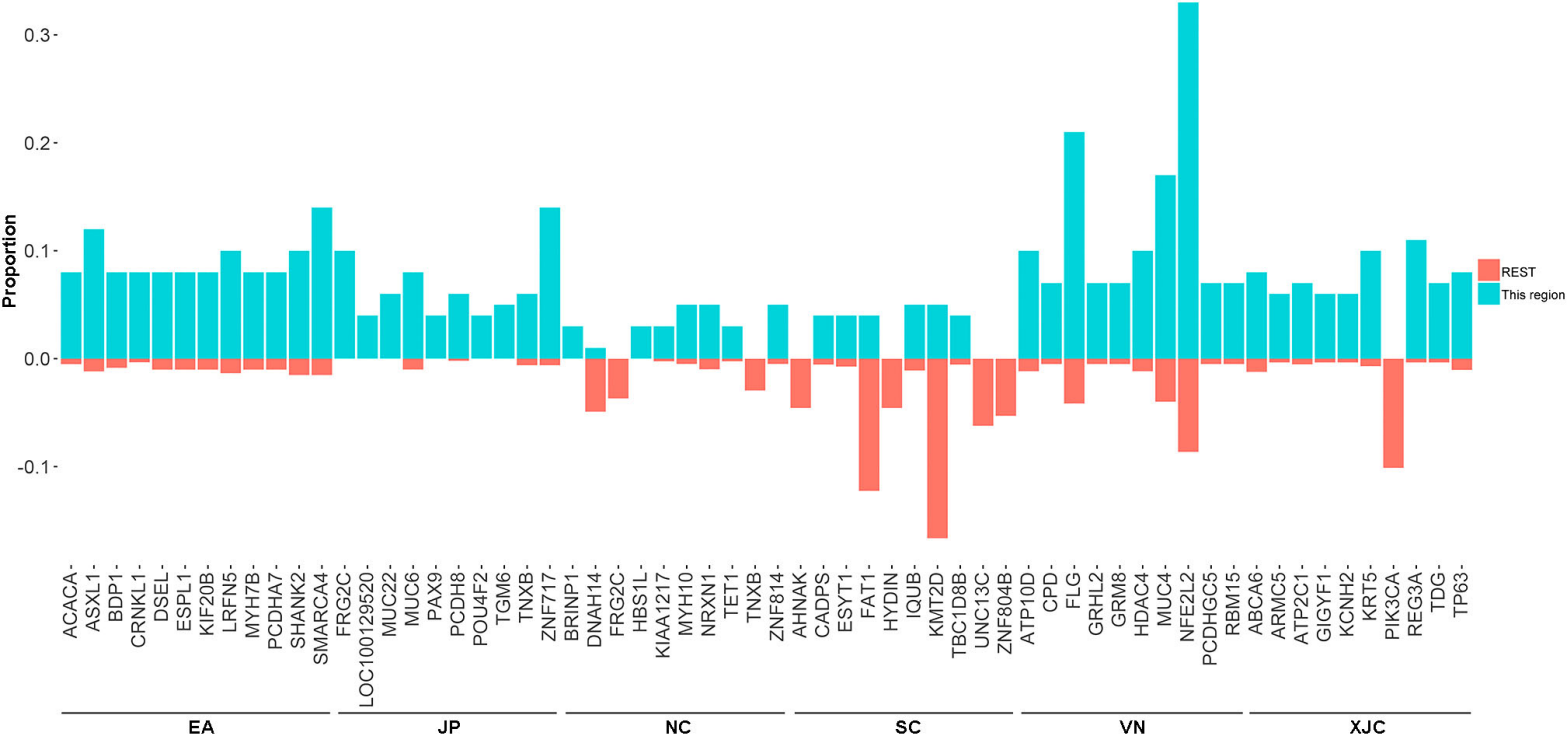
Proportion of the most enriched mutant genes (top 10) in patients with ESCC from different regions. NC, northern China; SC, southern China; XJC, Xinjiang, China; JP, Japan; EA, Europe and America; VN, Vietnam. REST: All regions, except for the included region; ESCC, esophageal squamous cell carcinoma.

The union set of the top 30 mutated genes in each group contained 96 genes, suggesting that the most commonly mutated genes in ESCC varied significantly across geographical regions (**Figures 4B**, **5**). The most enriched mutant genes in each region were *MYH10* and *ZNF814* in the NC group, *CADPS* and *TBC1D8B* in the SC group, *NFE2L2* in the VN group, *REG3A* in the XJC group, *SMARCA4* in the EA group, and *ZNF717* in the JP group (**Figure 5**). The rarest mutated genes were *FRG2C*, *KMT2D*, and *PIK3CA* in the NC, SC, and XJC groups, respectively (**Figure 5**).

**Figure 6:**
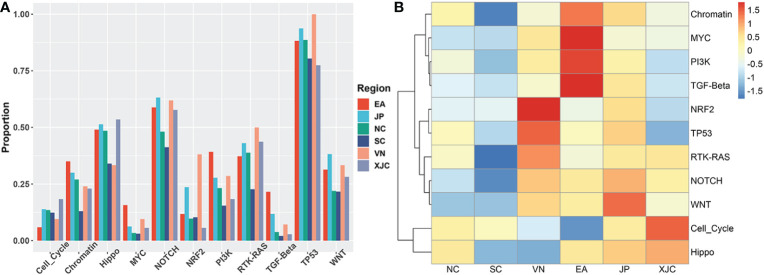
Geographical differences in signaling pathways. **(A)** Proportion of mutated samples in oncogenic signaling pathways in patients with ESCC from different regions. **(B)** Clustering analysis based on the proportion of mutant samples in oncogenic signaling pathways. NC, northern China; SC, southern China; XJC, Xinjiang, China; JP, Japan; EA, Europe and America; VN, Vietnam; ESCC, esophageal squamous cell carcinoma.

To explore the molecular mechanisms underlying the pathogenesis of ESCC in different regions, we further compared the differences in the fractions of mutated samples in 11 signaling pathways. The most altered pathways were the NRF2 and TP53 pathways in the VN group, MYC, PI3K, Chromatin, and TGF-Beta pathways in the EA group, WNT pathway in the JP group, and Cell Cycle and Hippo pathway in the XJC group (**Figure 6**).

### 3.2 Genomic analysis of ESCC between northern and southern China

**Figure 7:**
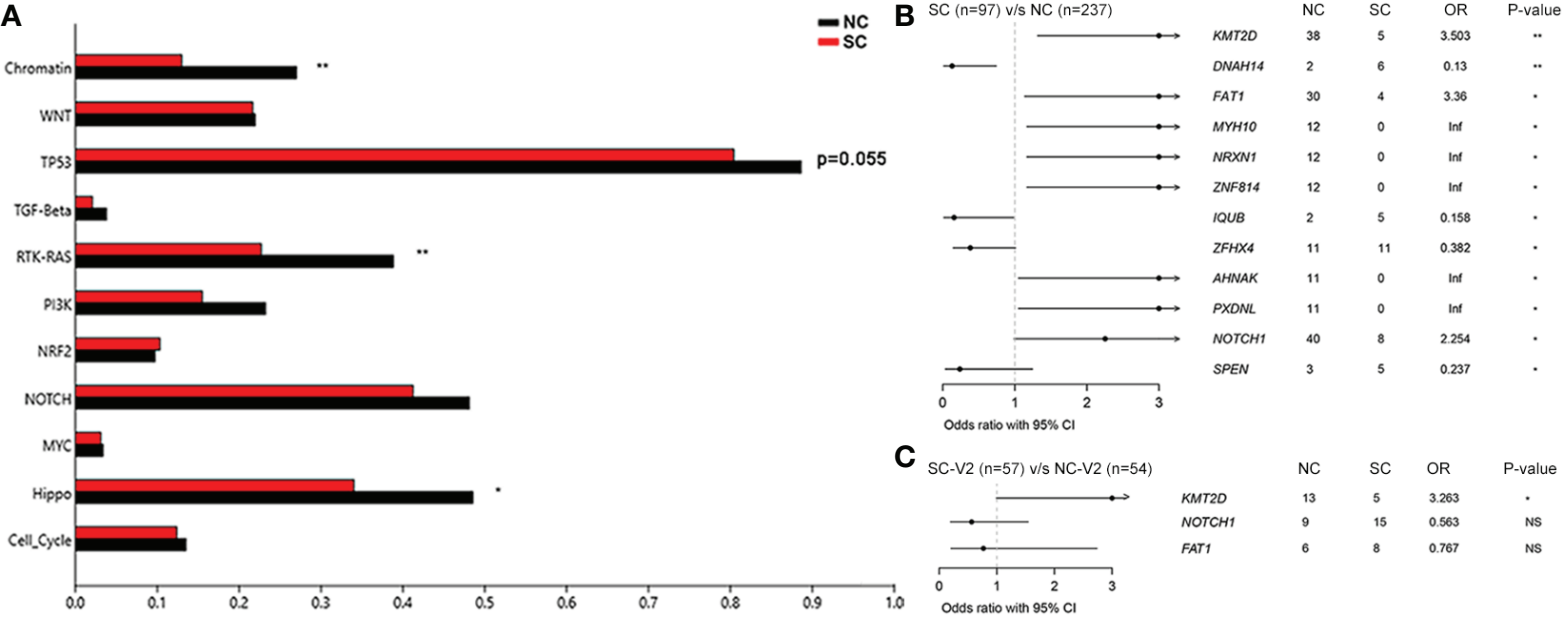
Genomic analysis of ESCC between northern and southern China. **(A)** Comparison of the fraction of mutated samples in oncogenic signaling pathways between the NC and SC groups in the discovery cohort. **(B)** Forest plot of genes with significantly different mutation frequencies between the NC and SC groups in the discovery cohort. **(C)** Validation of genes with significantly different mutation frequencies. Fisher’s exact test, *, P < 0.05; **, P < 0.01. NC, northern China; SC, southern China; NC-V2, northern China: validation set 2; SC-V2, northern China: validation set 2; ESCC, esophageal squamous cell carcinoma.

We further focused on the genomic differences in ESCC between patients from NC and SC. In the discovery set, the fraction of mutated samples in the three signaling pathways (Chromatin, RTK-RAS, and Hippo) in the NC group was significantly higher than that in the SC group (**Figure 7A**). Esophageal squamous cell carcinoma cases in the NC group were highly significantly associated with the presence of significantly mutated genes (SMGs) including *KMT2D*, *FAT1*, and *NOTCH1* mutations (**Figure 7B**). An additional 111 ESCC cases from NC and SC (termed NC-V2 and SC-V2, respectively) were sequenced and used as a validation set. The mutational landscapes of these cases are summarized in **Supplementary Figure 2**. In the validation set, *KMT2D* mutations were more frequent in the NC-V2 group (**Figure 7C**). Notably, the mutation data for this validation set were derived from the results of panel-targeted sequencing, and only three of the differential genes (*KMT2D*, *FAT1*, and *NOTCH1*) between the NC and SC groups in the discovery set were covered by this panel.

### 3.3 Geographical differences in genes associated with clinical features

**Figure 8:**
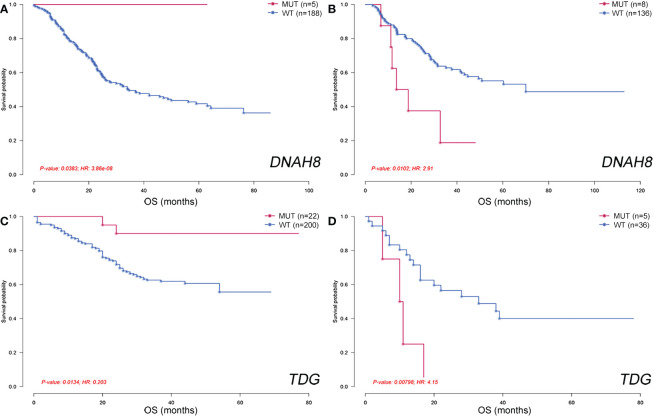
Geographical differences in genes associated with overall survival. Overall survival of patients stratified by *DNAH8* mutation status in the NC **(A)** and JP **(B)** groups. Overall survival of patients stratified by *TDG* mutation status in the NC-V2 **(C)** and XJC **(D)** groups. NC, northern China; XJC, Xinjiang, China; JP, Japan; NC-V2, northern China: validation set 2; OS, overall survival; MUT, mutated; WT, wild type; HR, hazard ratio.

Finally, we analyzed the geographical differences in the genes associated with clinical features, including smoking, drinking, and OS. Genes that were significantly associated with smoking or drinking in each group were screened using Fisher’s exact test. No smoking- or drinking-related genes were shared between the NC, SC, JP, and XJC groups (**Supplementary Figures 3A, B**). Prognosis-related genes were rarely shared among the four groups. Furthermore, some genes were correlated with OS quite differently in patients with ESCC from different geographical regions. Patients with mutated *DNAH8* had significantly better OS than patients with wild-type *DNAH8* in the NC group (**Figure 8A**; hazard ratio [HR] < 0.001, P = 0.038). However, the opposite pattern was observed in the JP group (**Figure 8B**; HR = 2.91, P = 0.010). Patients with mutated *TDG* also showed significantly better OS than those with the wild-type allele in patients with stage II ESCC in the NC-V2 group (**Figure 8C**; HR = 0.203, P = 0.013). However, the opposite pattern was observed in patients with stage II ESCC in the XJC group (**Figure 8D**; HR = 4.15, P = 0.008).

## 4 Discussion

Esophageal squamous cell carcinoma has distinct incidence, etiological risk factor, and survival characteristics in different regions worldwide (6). Although several studies have characterized the genomic characteristics of ESCC among countries or races, the results of genomic differences among different geographic regions are limited. In the present study, we performed an integrative analysis of ESCC among six geographic regions by integrating 1081 cases from previous reports and 111 cases from our cohort. In particular, we compared the genomic differences between patients with ESCC from NC, SC, JP, and XJC.

Previous studies have shown that the survival of patients with ESCC varies among countries (10, 15). To reduce the effect of stage, we compared OS between patients with stage II ESCC. Patients with ESCC from JP had the best OS, those from NC and SC had the second-best OS, and those from XJC had the worst OS. This difference may be related to differences in the DNA mutation signature. DNA mutation signature is associated with endogenous processes and exogenous mutagenic exposure (16, 17). Similar to findings of previous studies (22, 26), the overall mutation signatures of patients with ESCC were similar across regions. However, mutation signatures of COSMIC_3 and COSMIC_6 were detected in the XJC group, and those of COSMIC_2 and COSMIC_4 were observed in the VN group. COSMIC_3 and COSMIC_6 are associated with defective HR DNA repair and defective DNA mismatch repair, respectively (16, 17). COSMIC_4 was previously found to be associated with tobacco smoking (16, 17). In addition, COSMIC_2 (C > G at TpCpN trinucleotide) and COSMIC_13 (C > T at TpCpN trinucleotide) are driven by the activity of the APOBEC family of cytidine deaminases (16, 17). However, COSMIC_2 was detected in patients with ESCC from VN, whereas COSMIC_13 was detected in patients from other regions. The COSMIC_2 signature is associated with smoking and chewing tobacco (17). High TMB is also associated with smoking in squamous cell carcinoma (31). Therefore, high TMB values in the VN and XJC groups may be related to smoking and defects in DNA repair, respectively.

The APOBEC family can deaminate cytosine to uracil, leading to a cluster of mutations in various types of cancers (32). The APOBEC signature is a potential oncogenic pathway for mutational mechanisms in ESCC (21). The proportion of patients with ESCC enriched for the APOBEC signature was similar across regions. However, except for *ZNF750* and *RB1*, there were no other genes associated with APOBEC features common between patients with ESCC across regions.

The similarity of somatic mutation characteristics of patients with ESCC from different regions was analyzed using DNA mutation signatures and mutation profiles of the most common genes. The mutation characteristics of ESCC were most similar between NC and SC. Patients with ESCC from JP and XJC were similar to those from NC and SC, respectively. Notably, although VN is also located in East Asia, the differences in molecular characteristics between VN and Chinese patients with ESCC were greater than those between EA and Chinese patients with ESCC. A distinctive feature is that patients with ESCC from VN have the highest proportion of *NFE2L2* mutations and *NRF2* pathway alterations. *NFE2L2* (also known as *NRF2*) encodes a transcription factor that induces cellular responses to oxidative damage and plays an important role in ESCC development (33). Previous reports suggest that *NFE2L2* mutations are enriched in Asian patients with ESCC (23, 34), but this phenomenon may be confined to patients with ESCC from VN.

Next, we sought to understand the differences in the pathways involved in the development and progression of ESCC among regions. Genes involved in the NRF2 and TP53 pathways were more altered in the VN group, whereas genes involved in the Cell Cycle and Hippo pathways were more altered in the XJC group. *NFE2L2* is also associated with poor prognosis and resistance to chemoradiotherapy (35). A recent study suggested that ESCC subtypes with upregulated cell cycle-related genes were associated with the worst OS (36). In addition, *TDG* mutations were associated with a better prognosis in patients with ESCC from NC, but a worse prognosis in patients from XJC.

Although the mutation characteristics of ESCC were similar between patients from NC and SC, differences in SMGs were also observed. Significantly mutated genes, including *KMT2D*, *FAT1*, and *NOTCH1* were frequently identified in patients from NC compared to patients from NC. We further confirmed the high mutation frequency of *KMT2D* in NC compared to SC using targeted sequencing data from the validation set, which is consistent with the findings of a previous report (18). *KMT2D* encodes a conserved protein of the SET1 family of histone lysine methyltransferases and is a tumor-suppressor in ESCC (18). Chinese patients with ESCC with *KMT2D* mutations are significantly associated with high TMB (37). Compared to patients with ESCC from SC, a high TMB was also observed in patients with NC.

This study had some limitations. First, all patients with ESCC in the NC and SC groups were Han Chinese, whereas patients with ESCC in the XJC group were Kazakh Chinese. Different ethnicities may have influenced the findings on the regional differences in our study. Unfortunately, this limitation is not well addressed by currently published data. Second, relevant clinical information was not available for our cohort. Third, the mutation data in our cohort were based on target sequencing, which missed several important genes and pathways. These limitations prevented us from performing further validation in our cohort. Finally, it is important to note that survival analysis is influenced by treatment, stage, and other factors. The variability in these factors across cohorts may have affected the reliability of the relevant findings. In conclusion, our data suggest that ESCC is shaped by complex mutational mechanisms that vary among geographic regions. These differences may be related to differences in environmental carcinogens, incidence, and survival. Understanding these differences is important for tumorigenesis and personalized treatment.

## Data availability statement

The datasets presented in this study can be found in online repositories. The names of the repository/repositories and accession number(s) can be found in the article/**Supplementary Material**.

## Ethics statement

The studies involving human participants were reviewed and approved by Cancer Hospital Chinese Academy of Medical Sciences. The patients/participants provided their written informed consent to participate in this study.

## Author contributions

RZ, CL, and ZhiW contributed equally to this study. RZ,YiL, and JH designed the study and reviewed the manuscript. YiL and JH obtained financial support and oversaw the study. CL, JQ, YoL, QZ, ZheW, XK, and XC collected the samples and clinical data. ZhiW and YuL performed sequencing and statistical analyses. RZ, CL, and ZhiW analyzed the results and drafted the manuscript. The authors read and approved the final manuscript.
