# Supplementary material for: Comparative genomic analysis of esophageal squamous cell carcinoma among different geographic regions
Source: Front Oncol. 2023 Jan 18;12:999424. doi: 10.3389/fonc.2022.999424 (PMC9889985; doi:10.3389/fonc.2022.999424)
Supplement: Supplementary file 1 [file DataSheet_1.docx]

Supplementary Material

## Supplementary Figures


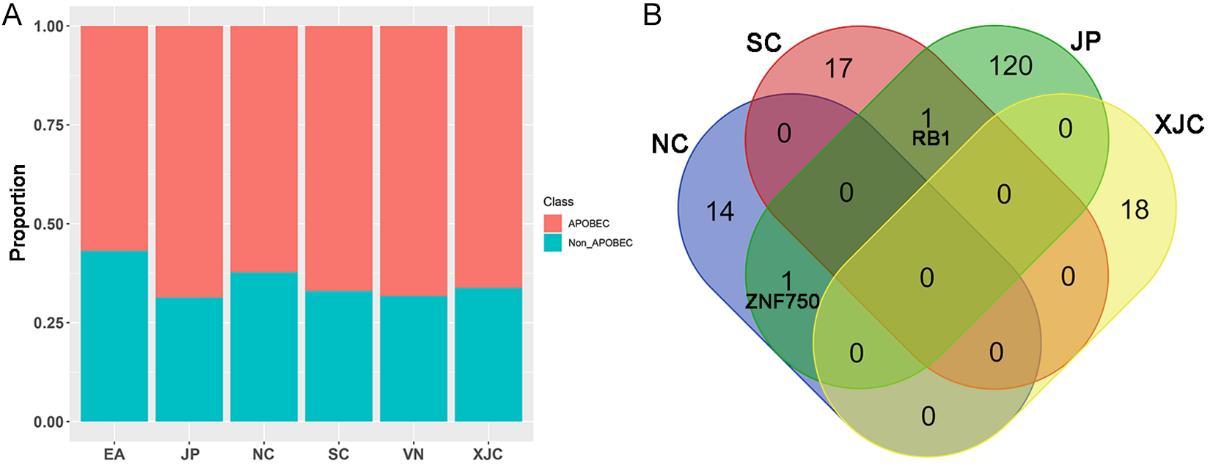


**Supplementary Figure 1.** Analysis of APOBEC signature of patients with ESCC from different regions. (A) Proportion of APOBEC signature enriched patients with ESCC. (B) Venn plot of APOBEC signature-related genes in patients with ESCC from different regions. NC, northern China; SC, southern China; XJC, Xinjiang, China; JP, Japan; EA, Europe and America; VN, Vietnam; ESCC, esophageal squamous cell carcinoma.


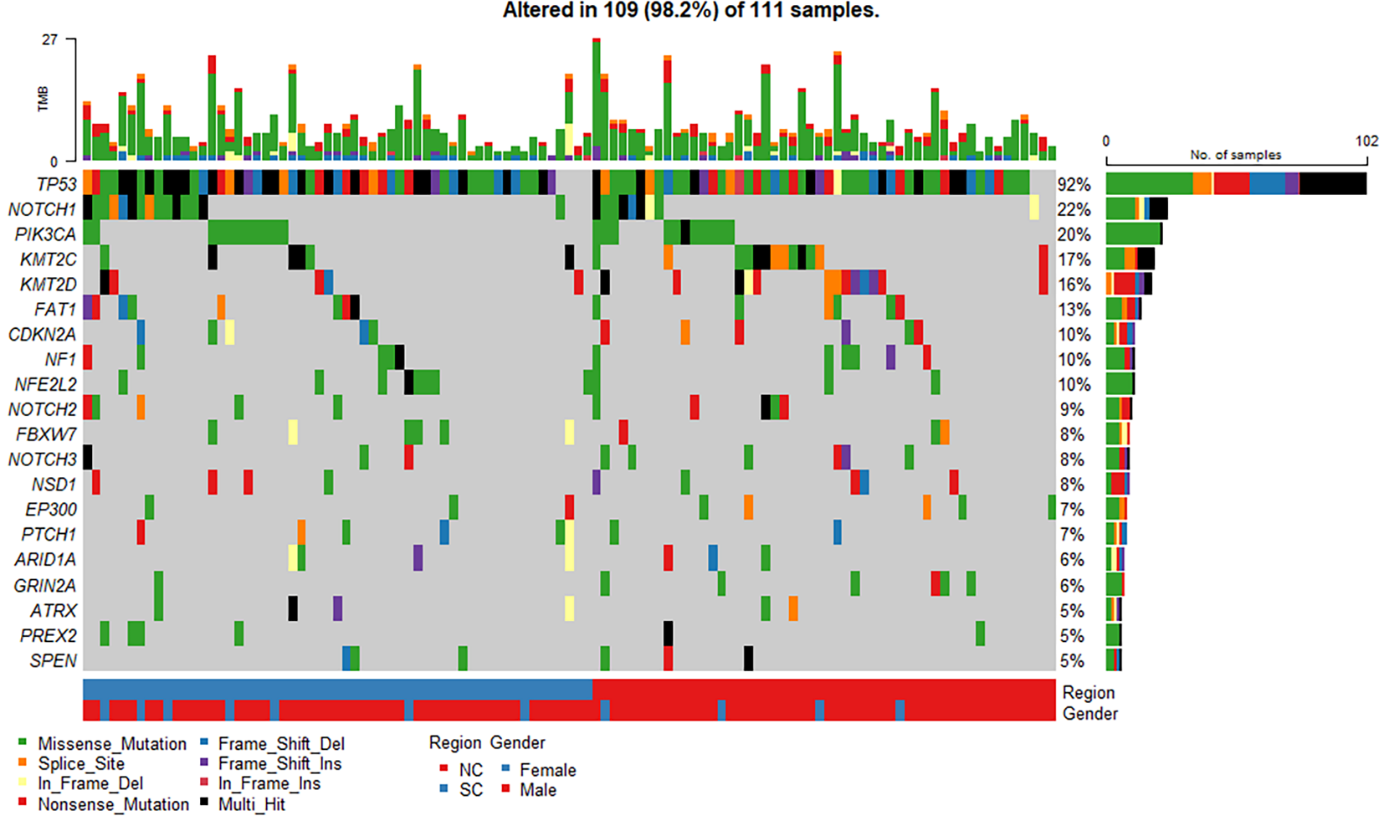


**Supplementary Figure 2.** Mutational landscape of our ESCC cohort (top 20 genes). The genes were sorted by mutation frequency, whereas samples were sorted by region. NC, northern China; SC, southern China; ESCC, esophageal squamous cell carcinoma.


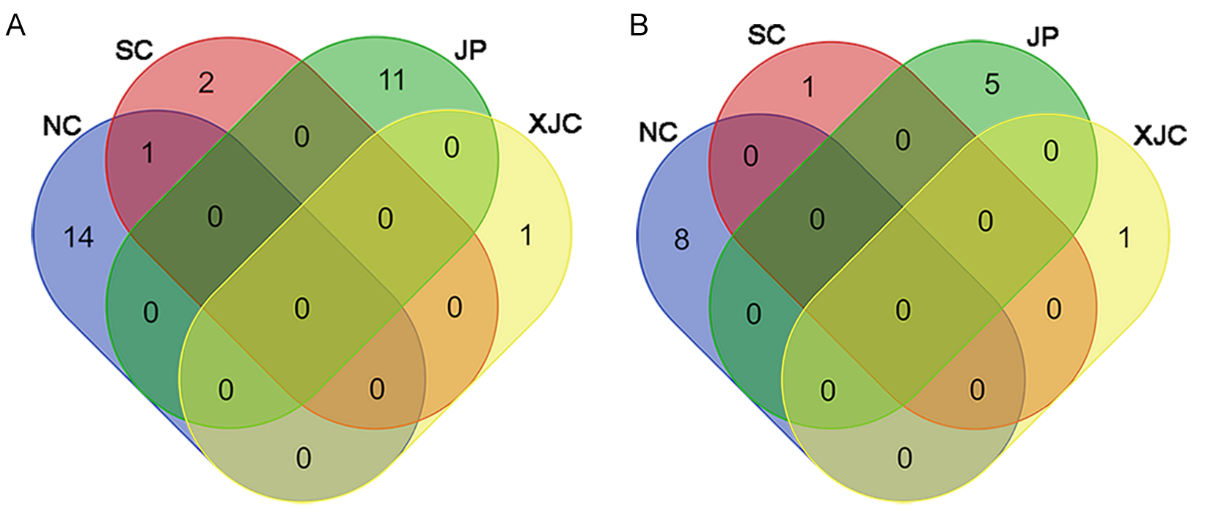


**Supplementary Figure 3**. Venn plot of smoking (A) or drinking (B) related genes in patients with ESCC from different regions. NC, northern China; SC, southern China; XJC, Xinjiang, China; JP, Japan; ESCC, esophageal squamous cell carcinoma.
